# Sparse coding model captures V1 population response statistics to natural movies

**DOI:** 10.1186/1471-2202-14-S1-P334

**Published:** 2013-07-08

**Authors:** Mengchen Zhu, Ian Stevenson, Urs Köster, Charles M Gray, Bruno A Olshausen, Christopher J Rozell

**Affiliations:** 1 Department of Biomedical Engineering, Georgia Institute of Technology, Atlanta, GA 30332, USA; 2 Redwood Center for Theoretical Neuroscience, University of California, Berkeley, Berkeley, CA 94720, USA; 3 Cell Biology and Neuroscience, Montana State University, Bozeman, MT 59717, USA; 4 School of Electrical and Computer Engineering, Georgia Institute of Technology, Atlanta, GA 30332, USA

## 

Local populations of sensory cortical cells exhibit a diverse range of activity patterns. However, classical approaches have neither fully accounted for nor characterized this heterogeneity, especially in response to natural stimuli. First, classical single cell recordings suffered from sampling bias and favored highly responsive cells [1]. Second, common approaches considered mostly the average activity over different cell classes, without a full description of the statistical distribution over the entire population [2]. Recent studies started to address these issues [3,4]. In this study, we make further inroads by recording simultaneous single unit activities across cortical layers in cat V1 in response to natural movies using a silicon polytrode, and comparing the population statistics to the predictions from a dynamical system implementation of the sparse coding model [5,6], with a linear-nonlinear model as control.

We analyzed data sets from two recording sessions in anaesthetized cats viewing natural movies. To quantitatively measure the difference between model predictions and the recording, we used the earth mover's distance [7] to quantify the dissimilarities between the distributions of the recorded response and those predicted by sparse coding and linear-nonlinear model. We show that: (1) The population firing rate distribution is close to exponential in both the recorded data and the sparse coding model in response to natural movies; (2) The response correlation between unit activities is small regardless of the size of receptive field overlap, when using a binning window synced to the movie frame. A similar relationship between the response correlation and receptive field overlap is observed in the sparse coding model; (3) A linear-nonlinear model could predict the exponential firing rate distribution, but not the correlation structure.

## References

[B1] Olshausen B Field D How close are we to understanding V1? Neural Computation 2005 17 8 1665 1699 10.1162/0899766054026639 15969914

[B2] Wohrer A Humphries MD Machens C Population-wide distributions of neural activity during perceptual decision-making Progress in Neurobiology 2012 23123501 10.1016/j.pneurobio.2012.09.004 PMC5985929

[B3] Yen S Baker J Gray C Heterogeneity in the responses of adjacent neurons to natural stimuli in cat striate cortex Journal of neurophysiology 2007 97 2 1326 17079343 10.1152/jn.00747.2006

[B4] Herikstad R Baker J Lachaux JP Gray CM Yen SC Natural Movies Evoke Spike Trains with Low Spike Time Variability in Cat Primary Visual Cortex The Journal of Neuroscience 2011 31 44 15844 15860 10.1523/JNEUROSCI.5153-10.2011 22049428 PMC6623039

[B5] Olshausen B Field D Emergence of simple-cell receptive field properties by learning a sparse code for natural images Nature 1996 381 6583 607 609 10.1038/381607a0 8637596

[B6] Rozell C Johnson D Baraniuk R Olshausen B Sparse coding via thresholding and local competition in neural circuits Neural Computation 2008 20 2526 2563 10.1162/neco.2008.03-07-486 18439138

[B7] Rubner Y Tomasi C Guibas L The Earth Mover's Distance as a Metric for Image Retrieval International Journal of Computer Vision 2000 40 99 121 10.1023/A:1026543900054

